# Prevalence and antibiotic resistance of rectal Mollicutes in HIV-infected men who have sex with men at the University Hospital of Dresden, Germany

**DOI:** 10.1007/s15010-019-01386-3

**Published:** 2020-01-28

**Authors:** Petra Spornraft-Ragaller, Roger Dumke

**Affiliations:** 1 Klinik und Poliklinik für Dermatologie, Universitätsklinikum Carl Gustav Carus, Technische Universität Dresden, Dresden, Germany; 2 Institut für Medizinische Mikrobiologie und Hygiene, Technische Universität Dresden, Dresden, Germany

**Keywords:** Sexually transmitted infection, Ureaplasma, Mycoplasma, Resistance, HIV, MSM

## Abstract

**Background:**

Rectal sexually transmitted infections (STI) are common in men having sex with men (MSM). *Mycoplasma genitalium* is increasingly being reported in this localization, but due to frequent lack of symptoms at this site, clinical significance is still unclear. Rectal prevalence of *Mycoplasma hominis* and *Ureaplasma* species is not well studied so far. We aimed to investigate the prevalence and antibiotic sensitivity of rectal Mollicutes in our HIV-cohort.

**Methods:**

In 227 MSM presenting for annual STI-screening, 317 anorectal swabs were collected from January 2017 to December 2018. PCR was performed for detection of *Chlamydia trachomatis*, *Neisseria gonorrhoeae*, *M. genitalium* and also culture for *M. hominis* and *Ureaplasma* spec.

**Results:**

Prevalence for *M. genitalium*, *M. hominis*, *Ureaplasma* spec., *C. trachomatis* and *N. gonorrhoeae* was 8.2%, 7.3%, 12.0%, 5.1% and 1.9%, respectively. Patients were asymptomatic with few exceptions. Seroprevalence of syphilis in 227 MSM was 41.9%. In 20 strains of *M. genitalium*, resistance-associated mutations to macrolides and quinolones were found in 60% and 30%, respectively; in five strains (25%) to both. *M. hominis* and *Ureaplasma* spec. frequently occurred combined, mostly in significant quantity consistent with infection. *M. hominis* and *Ureaplasma* spec. regularly showed sensitivity to tetracycline.

**Conclusion:**

At screening, rectal colonization with Mollicutes was common in our patients, but rarely caused symptoms. Due to rising antibiotic resistance of *M. genitalium* against quinolones, therapeutic options are increasingly limited. Treatment should be guided by antibiotic resistance testing including quinolones. In persisting anorectal symptoms, *M. hominis* and *Ureaplasma* spec. should also be taken into account.

## Introduction

Rectal sexually transmitted infections (STI) are common in men having sex with men (MSM) and not restricted to *Chlamydia trachomatis* or *Neisseria gonorrhoeae*. Especially the cell wall-less members of the class Mollicutes have been discussed as potentially symptom- and/or transmission-relevant bacteria. The extremely slow-growing species *Mycoplasma genitalium* is a known pathogen for nongonococcal urethritis in males and associated with cervicitis and pelvic inflammatory disease in women [1–4]. It is increasingly found in rectal swabs of MSM as well, but due to predominantly asymptomatic infections in this localization, clinical significance is still unclear. Among African women, *M. genitalium* is reported to facilitate HIV-infection [5, 6], but comparable studies in MSM are lacking. Treatment of *M. genitalium* infections is hampered by intrinsic resistance to betalactams, frequent occurrence of macrolide resistance and limited clinical efficiency of doxycycline. Reports on increasing quinolone resistance mainly from the Asian Pacific region currently raise concerns of spreading of multiresistant strains with strongly limited treatment options [7]. Rectal prevalence and clinical significance of *Mycoplasma hominis* and *Ureaplasma* spec. are not well studied so far. We aimed to investigate the prevalence of rectal STI and Mollicutes as well the antibiotic sensitivity of mycoplasma and ureaplasma species in our cohort of HIV-positive homosexual men.

## Methods

In 227 HIV-infected MSM presenting for annual STI-screening, 317 anorectal swabs were collected from January 2017 to December 2018. In some patients, two swabs were available. DNA in each sample was extracted using the EZ1 automatic system (QIAGEN) and commercial real-time PCR approaches were performed for detection of *N. gonorrhoeae*, *C. trachomatis*, *M. genitalium* (TIB Molbiol) according to the recommendations of the manufacturer. In parallel, culture for propagation of *M. hominis* and *Ureaplasma* spec. was carried out. Molecular resistance testing for *M. genitalium* was done by partial amplification of 23S rRNA (macrolide resistance) and *parC* gene (quinolone resistance) and sequencing as described [8]. Mycoplasma IST test (BioMerieux) was used for cultivation and phenotypical resistance testing of *M. hominis* and *Ureaplasma* spec. Symptoms were evaluated at routine clinical follow up, without using a questionnaire. In all patients, seroprevalence of syphilis (TPHA > 1:80, VDRL > 1:2) was determined. The study was approved by the ethical committee of the TU Dresden (no 189062009).

For statistical analysis, Fisher’s exact test for analysis of categorial data was performed. Because the Chi^2^ assumption of five or more cases in one row of the contingency table is violated for *N. gonorrhoeae*, Fischer’s exact test (two-sided) was used and calculated by Stata Version 15.1.

Statistical significance is considered at a *p* value of ≤ 0.05.

## Results

**Fig. 1:**
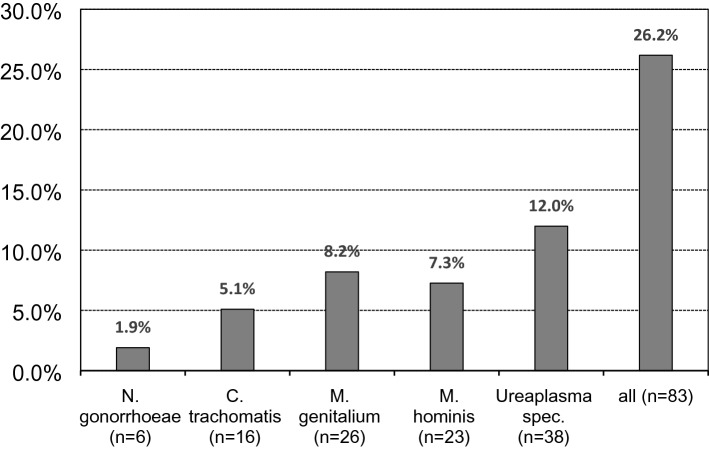
Prevalence of STI in 317 anorectal swabs of 227 HIV-positive patients

Of 317 anorectal swabs, 83 (26%) were positive for at least one STI. Prevalence for *M. genitalium*, *M. hominis*, *Ureaplasma* spec., *C. trachomatis* and *N. gonorrhoeae* was 8.2%, 7.3%, 12.0%, 5.1%, and 1.9%, respectively (Fig. 1). Seroprevalence of syphilis in 227 MSM (mean age 40.4 years, median age 47 years) was 41.9%, but in statistical analysis this was not significantly associated with a distinctive anal coinfection. 15 patients were identified with a VDRL > 1:2 indicating prevalent or recent active syphilis, of whom only four had a positive rectal swab with the STI mentioned above. However, MSM with a syphilis-positive status had a 47.1% increased risk of requiring at least one additional infection compared to those with a syphilis-negative status; the highest relative risk was found for *N. gonorrhoeae* (Table 1). Although none of the associations reached statistical significance on a 5% alpha level, this is probably due to the size of the unselected sample.

**Table 1:** Association of syphilis-seropositive patients with detection of *N. gonorrhoeae, C. trachomatis, M. genitalium, M. hominis,* and *Ureaplasma* spec. in rectal swabs of HIV-positive MSM

Syphilis-positive (*n* = 95)	%	Syphilis-negative (*n* = 132)	%	*p* value	RR (95% CI)
All (*n* = 36)	37.9	All (*n* = 34)	25.8	0.059	1.471 (0.999–2.168)
*N. gonorrhoeae* (*n* = 5)	5.3%	*N. gonorrhoeae* (*n* = 1)	0.8%	0.085	6.947 (0.825–58.507)
*C. trachomatis* (*n* = 5)	5.3%	*C. trachomatis* (*n* = 10)	7.6%	0.594	0.695 (0.245–1.967)
*M. genitalium* (*n* = 11)	11.7%	*M. genitalium* (*n* = 12)	9.1%	0.657	1.274 (0.587–2.763)
*M. hominis* (*n* = 10)	10.5%	*M. hominis* (*n* = 10)	7.6%	0.482	1.389 (0.602–3.205)
*Ureaplasma* spec. (*n* = 19)	20.0%	*Ureaplasma* spec. (*n* = 15)	11.4%	0.090	1.760 (0.943–3.283)

**Fig. 2:**
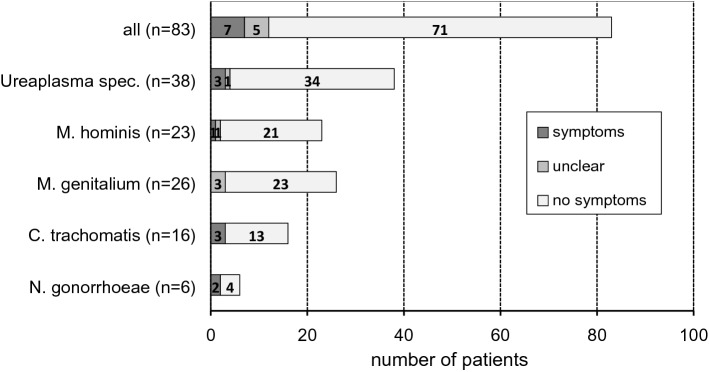
Occurrence of symptoms in 83 patients tested positive for STI

Most patients with anorectal STI positive swabs were asymptomatic (85.5%), some even in presence of *N. gonorrhoeae* and *C. trachomatis*. Symptoms were most frequently associated with detection of *N. gonorrhoeae* (2 of 6, 33.3%) and *C. trachomatis* (3 of 16, 18.8%) (Fig. 2). In 22 (26.5%) swabs, more than one STI was detected and in four swabs (4.8%) at least three different STI were found. Particularly *M. genitalium* (84.6% of positive swabs) and to a lesser extent *Ureaplasma* spec., were detected as single infection, whereas in more than half of *M. hominis*-positive swabs other STI were found as well, predominantly *Ureaplasma* spec. (Table 2).

**Table 2:** Occurrence of Mollicutes species in 317 rectal swabs of HIV-positive MSM

Species	Number of positive samples (%)
*M. genitalium*	22 (6.9)
*M. genitalium* + *M. hominis*	0
*M. genitalium* +* Ureaplasma* spec.	2 (0.6)
*M. genitalium* +* M. hominis* +* Ureaplasma* spec.	2 (0.6)
*M. genitalium* + *C. trachomatis*	0
*M. genitalium* +* N. gonorrhoeae*	0
*M. hominis*	8 (2.5)
*M. hominis* +* Ureaplasma* spec.	9 (2.8)
*M. hominis* +* N. gonorrhoeae*	2 (0.6)
*M. hominis* +* Ureaplasma* spec. +* C. trachomatis*	1 (0.3)
*M. hominis* +* Ureaplasma* spec. +* C. trachomatis* +* N. gonorrhoeae*	1 (0.3)
*Ureaplasma* spec.	19 (6.0)
*Ureaplasma* spec. + *C. trachomatis*	4 (1.3)
*Ureaplasma* spec. + *N. gonorrhoeae*	0

**Fig. 3:**
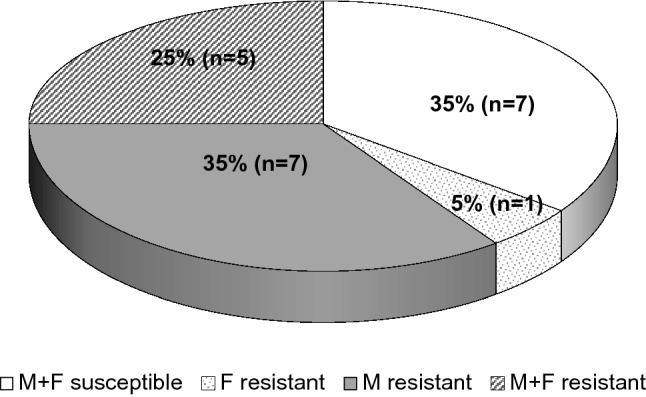
Occurrence of mutations associated with macrolide (M) and fluorochinolone (F) resistance among 20 *M. genitalium* strains

Of 25 *M. genitalium*-positive samples, in 20 strains amplification products of targets 23S rRNA and *parC* could be obtained of which 60% and 30% showed resistance-associated mutations to macrolides and quinolones, respectively. Five strains (25%) exhibited combined resistance to both macrolides and quinolones (Fig. 3). Most frequent (75%) single-nucleotide polymorphism (SNP) in the 23S rRNA linked to macrolide resistance was located at position 2059 (*E. coli* numbering, base exchange adenine to guanine) whereas an A–G transition at position 2058 was confirmed in the remaining three strains. Most frequent mutation in the *parC* gene associated with quinolone resistance was found at position 248 (four of six strains) leading to an amino acid change from serine to isoleucine (aa 83). Different changes were confirmed at positions 81 (glycine–cysteine) and 87 (aspartic acid to asparagine) in the quinolone-resistance determining region (QRDR) of two strains.

In 46 of 48 cultures, antibiotic sensitivity of *M. hominis* and *Ureaplasma* spec. was determined. Overall, 23 cultures of *Ureaplasma* spec. and 10 cultures of *M. hominis* were available. In 13 mixed cultures, material was not sufficient for differentiation between both species. *Mycoplasma hominis* and *Ureaplasma* spec. frequently occurred combined and colony forming units in culture were > 10^4^ in most patients, of whom some exhibited symptoms. Interestingly, in one patient complaining of anal discomfort, *U*. *urealyticum* (in this case, strain was typed by PCR, data not shown) was the only pathogen detected and the patient responded clinically to subsequent therapy with doxycycline. *Ureaplasma* spec. and *M. hominis* strains regularly showed sensitivity to tetracycline. As expected, all *M. hominis* strains were resistant against erythromycin, azithromycin, clarithromycin and most *Ureaplasma* spec. (20/23) against ciprofloxacin. Occurrence of tetracycline-resistant *M. hominis* and *Ureaplasma* spec. as well macrolide-resistant *Ureaplasma* spec. strains was not confirmed.

## Discussion

In a screening situation, rectal colonization with Mollicutes was more common than with “classical” STI’s in HIV-infected MSM investigated in our center. In this respect, the most frequently detected potential STI from the Mollicutes class was *Ureaplasma* spec. (12.0%), followed by *M. genitalium* (8.2%) and *M. hominis* (7.3%), respectively. Symptoms were rare but when occurred, they seemed to be more related to *Ureaplasma* spec. than to *M. hominis* or *M. genitalium*. However, even confirmed *N. gonorrhoeae* and *C. trachomatis* infections were symptomatic in only 33% and 19% of cases, respectively. Rectal prevalence of *N. gonorrhoeae* was relatively low in our cohort, however, rectal detection of *C. trachomatis* (5.1%) was equivalent to a British study screening MSM for STI (5.6%) [9]. Seroprevalence of syphilis was comparable to the population of MSM enrolled in the German HIV seroconverter study group (39.6%); however, these were younger (median 33 years) than our patients [10]. Due to the relatively small cohort, we not able to find a statistical significant association of syphilis seroprevalence and other rectal STI in HIV-positive MSM, but those with a syphilis-positive status had a 47.1% increased risk of acquiring at least one additional infection. Thus, syphilis may represent a proxy for increased sexual risk behaviour, leading to other STI as well.

The clinical significance of rectal Mollicutes detection is still unclear. *M. genitalium* is a known pathogen in nongonococcal urethritis (NGU), and suspected as involved in cervicitis and pelvic inflammatory disease [3, 11, 12]. It is most frequently found in men with symptomatic urethritis [13] with prevalence rates of 15–25% [1, 11]. In the general population, the prevalence is estimated from 1.1 to 3.9% [14, 15]. The microorganisms have been also detected in the anorectum of MSM where the bacteria may act as a reservoir for NGU. In a genitourinary medicine clinic in London, rectal prevalence in MSM was 4.4% and in HIV-infected MSM 14.1% [9]. In our patients, rectal prevalence of *M. genitalium* was 8.2%, which was higher than in a large German laboratory investigation on approximately 7500 patients, predominantly MSM, with a rectal prevalence of *M. genitalium* of 6.7% [16] and in another German study in HIV-positive MSM attending anal cancer screening (3.3%) [17]. Recent Australian studies reported rectal prevalence rates of *M. genitalium* in MSM of 7% and 8.9%, respectively [2, 18]. An infection in this localization is described to be mainly asymptomatic [19], but the role of *M. genitalium* as a causative agent for proctitis is discussed controversially [2, 9, 20, 21]. In an Australian study, rectal detection of *M. genitalium* (7.0% overall) was not more frequent in patients with symptoms of proctitis than in those without [18]. In our patients as well, no clear symptoms were associated with detection of *M. genitalium*. This is comparable with the results of a recent German study among MSM (around 50% HIV-positive), confirming anorectal symptoms in 8% of *M. genitalium*-positive patients [22]. Rectal and pharyngeal detection of *M. genitalium* is considered to be usually asymptomatic and thus, laboratory testing in these sites is not indicated in the general population. However, anal sampling of *M. genitalium* in MSM is epidemiologically important as most of infections could be missed [23] and might be transmitted if not treated.

Unfortunately, treatment of *M. genitalium* is increasingly complicated by the circulation of macrolide and quinolone-resistant strains worldwide. In our study, 60% and 30% of 20 strains showed mutations associated with macrolide and quinolone resistance, and 25% with both. Prevalence of macrolide resistance of *M. genitalium* may now exceed 50% [3, 24] and is reported to be even higher in HIV-positive patients [25] or HIV-PrEP users [26, 27], which corresponds to our results. The study from Germany calculated a rate of 80% macrolide-resistant strains [22]. Quinolone resistance and treatment failures are being reported especially from the Asian Pacific region [28–30] reaching nearly 90% in China [31]. So far, data from Europe on quinolone resistance are relatively limited. Results of recent studies from England, France, Russia/Estonia, Scandinavian countries and Spain demonstrated rates of quinolone resistance of 5%, 6%, 6%, 7%, and 8%, respectively [32–36]. Among MSM in Ireland, 33% of *M. genitalium* strains are quinolone-resistant [37] and in the above-mentioned German investigation, a rate of 13% was calculated [22]. Further studies have to clear, whether the different amino acid changes described in the QRDR of ParC influence the susceptibility of *M. genitalium* strains to quinolones. In recent reports, the common Ser83Ile transition was not associated with treatment failure after moxifloxacin therapy but phenotypic resistance testing resulted in significantly elevated MICs for different quinolones [38, 39].

In addition, in the anorectum of some of our patients, the urethral commensals *Ureaplasma* spec. and *M. hominis* could be detected by culture, mostly in significant quantity consistent with infection. Little is known on their pathogenic significance at this site and only few studies are available. In a recent Chinese study on 183 MSM, rectal prevalence of *M. hominis* detected by multiplex PCR was 7.1% [40], which is comparable with our results using culture (7.3%). Detection rates by PCR were much higher in a study from Ireland on 107 rectal swabs from MSM: 24.3% *U. urealyticum*, 24.3% *M. hominis*, and 4.7% *U. parvum*, respectively [41]. Symptoms in our patients were rare and if present, mainly found in presence of *Ureaplasma* spec.. Of note, in one of the symptomatic patients only *U*. *urealyticum* was detected. This may point to a possible pathogenic significance of these bacteria under certain conditions. However, as detailed description of clinical symptoms and test of cure is missing, a clear correlation between clinical picture and detection of *U. urealyticum* in this patient cannot be established. If using the Mycoplasma IST test, determination of resistance of the isolated *Ureaplasma* spec. and *M. hominis* strains against tetracycline and macrolides provided reliable data whereas the high rate of quinolone resistance must be questioned [42]. Overall, recent European studies reported also relatively low to moderate resistance rates (< 15%) of all three species to these antibiotic classes [43, 44].

Our study has several limitations: even when our HIV-cohort is still the largest in the region of Eastern Saxony, some patient selection bias can not be excluded. Anorectal symptoms were not evaluated by a questionnaire, but on clinical follow-up. Thus, some symptoms might have gone unnoticed; on the other hand, this corresponds to the normal clinical situation. Further, we did not evaluate complete antibiotic treatment, e.g., from outside of our center, so that antibiotic resistance could not be assigned to previous therapy. However, the results of the study confirm the frequent detection of members of the Mollicutes class in rectal samples of HIV-positive patients.

## Conclusion

Among the sexually transmitted Mollicutes species, public health importance of *M. genitalium* is now increasingly discussed. In current European guidelines, azithromycin is recommended as first-line treatment and moxifloxacin considered being still highly effective in Europe [4]. In the meantime, this might be doubtful in an increasing number of patients. Regarding multiresistant *M. genitalium* strains, only few alternative therapy regimens with little evidence of efficacy are available. Thus, whether asymptomatic patients or partners should be treated to minimize reservoirs for transmission of *M. genitalium* to susceptible persons has to be discussed in the light of limited treatment options and also of recent warnings of quinolone toxicity. According to our findings, we strongly recommend resistance testing whenever possible, not only against macrolides, but against quinolones as well. To avoid induction of resistance in other species, an ongoing transmission of the pathogen and unnecessary costs, results should be available before starting therapy, especially in populations with high prevalence of *M. genitalium* such as MSM. In persisting anorectal symptoms and exclusion of well-characterized STI pathogens, *M. hominis* and *Ureaplasma urealyticum* should also be taken into account.
